# Identifying the Implementation Conditions Associated With Positive Outcomes in a Successful Nursing Facility Demonstration Project

**DOI:** 10.1093/geront/gnaa041

**Published:** 2020-04-30

**Authors:** Susan E Hickman, Edward J Miech, Timothy E Stump, Nicole R Fowler, Kathleen T Unroe

**Affiliations:** 1 Indiana University Center for Aging Research, Regenstrief Institute Incorporated, Indianapolis; 2 Department of Community and Health Systems, School of Nursing, Indiana University, Indianapolis; 3 Center for Health Services Research, Regenstrief Institute Incorporated, Indianapolis, Indiana; 4 Department of Emergency Medicine, Indiana University School of Medicine, Indianapolis; 5 Department of Biostatistics, Indiana University School of Medicine, Indianapolis; 6 Department of General Internal Medicine and Geriatrics, Indiana University School of Medicine, Indianapolis

**Keywords:** Configurational analysis, Nursing home, Potentially avoidable hospitalizations, Quality, Transfers

## Abstract

**Background and Objectives:**

To identify the implementation barriers, facilitators, and conditions associated with successful outcomes from a clinical demonstration project to reduce potentially avoidable hospitalizations of long-stay nursing facility residents in 19 Indiana nursing homes.

**Research Design and Methods:**

Optimizing Patient Transfers, Impacting Medical quality, Improving Symptoms—Transforming Institutional Care (OPTIMISTIC) is a multicomponent intervention that includes enhanced geriatric care, transition support, and palliative care. The configurational analysis was used to analyze descriptive and quantitative data collected during the project. The primary outcome was reductions in hospitalizations per 1,000 eligible resident days.

**Results:**

Analysis of barriers, facilitators, and conditions for success yielded a model with 2 solution pathways associated with a 10% reduction in potentially avoidable hospitalizations per 1,000 resident days: (a) lower baseline hospitalization rates and investment of senior management; or (b) turnover by the director of nursing during the observation period. Conditions for success were similar for a 20% reduction, with the addition of increased resident acuity.

**Discussion and Implications:**

Key conditions for successful implementation of the OPTIMISTIC intervention include strong investment by senior leadership and an environment in which baseline hospitalization rates leave ample room for improvement. Turnover in the position of director of nursing also linked to successful implementation; this switch in leadership may represent an opportunity for culture change by bringing in new perspectives and viewpoints. These findings help define the conditions for the successful implementation of the OPTIMISTIC model and have implications for the successful implementation of interventions in the nursing facility more generally.

There is a growing focus on reducing potentially avoidable hospitalizations of nursing facility residents to improve the quality of care and reduce costs (Department of Health and Human Services—Office of Inspector General, 2013; Ouslander & Berenson, 2011). In 2012, the Centers for Medicare and Medicaid Services (CMS) issued a call for enhanced care coordination demonstration projects to reduce potentially avoidable hospitalizations of long-stay nursing facility residents. The OPTIMISTIC (Optimizing Patient Transfers, Impacting Medical quality, and Improving Symptoms—Transforming Institutional Care) project, designed and implemented by Indiana University and partners, was one of the seven funded sites nationally (Centers for Medicare and Medicaid Services, 2019).

OPTIMISTIC is a multicomponent clinical intervention designed to prevent potentially avoidable hospitalizations of long-stay nursing home residents (OPTIMISTIC, 2020; Unroe et al., 2015). The intervention is implemented by project registered nurses (RNs) who are embedded within participating nursing facilities to provide enhanced staff education and direct clinical care in a specially designed role. Project RNs are supported by nurse practitioners (NPs) who are also employed by the project. The clinical intervention includes enhanced geriatric care for acute and chronic conditions focused on the early identification and management of acute conditions and proactive chronic disease care as well as collaboration with primary care providers. Project RNs provide training and modeling in the use of tools to support communication created as part of the “Interventions to Reduce Acute Care Transfers” (INTERACT) model (INTERACT, 2020). A second component of the intervention model is a focus on safer transitions for residents who do transfer through improved communication. Following a resident transfer and return to the nursing facility, Project RNs complete a detailed root cause analysis using a modified INTERACT transfer tracking and quality improvement tool to identify contributing factors. Project NPs also conduct medication reconciliation and meet with the resident and family to provide education as well as review goals of care. The third component of the intervention model focuses on palliative care with an emphasis on advance care planning. All Project RNs and NPs are certified in the Respecting Choices Advanced Steps (Respecting Choices, 2017) facilitation model to support high-quality advance care planning and provided with structure to ensure every resident is given the opportunity to participate in advance care planning conversations (Hickman et al., 2016, 2019). Education about end-of-life care and symptom management was provided using the geriatric End-of-Life Nursing Education Consortium curriculum (Kelly, Ersek, Virani, Malloy, & Ferrell, 2008) and through consultation with a palliative care physician. Fidelity was monitored through an ongoing review of clinical outcomes data by the Medical, Transitions, and Palliative Care Cores responsible for oversight of each intervention component to identify and address variability in intervention delivery.

The OPTIMISTIC implementation process included meetings between project staff and the leadership of each of the 19 nursing facilities at the start of the project to provide information, answer questions, and identify an internal point person. Project RNs and NPs completed a 2-week intensive standardized training and were provided with an operations manual containing intervention procedures. During the first few weeks of implementation, Project RNs focused on becoming familiar with the facility and introducing OPTIMISTIC to staff and residents. Project RNs and NPs met regularly throughout the first year as a team and completed additional training on key project components (e.g., the Respecting Choices Advanced Steps facilitation model) that were then rolled out on the same timeline in each facility (Unroe et al., 2015).

In an external evaluation of the demonstration project, OPTIMISTIC successfully reduced all-cause hospitalizations by 25% and potentially avoidable hospitalizations by nearly 40% in comparison to a matched control group (Ingber et al., 2017). By design, the analysis combined the outcomes across participating OPTIMISTIC facilities and assessed outcomes by comparing between funded sites with matched controls. However, there was no evaluation of nursing facility-level data. In order to support dissemination of this successful model, it is important to identify which conditions (e.g., intervention elements, context) are most associated with the successful reduction of hospitalizations.

The configurational approach is a mathematical approach to identify combinations of conditions necessary or sufficient to achieve a specific, desired outcome (e.g., reduction in hospitalizations) (Baumgartner & Epple, 2014; Baumgartner & Thiem, 2015; Cragun et al., 2016; Kane, Lewis, Williams, & Kahwati, 2014). The configurational approach is fundamentally different than traditional correlation- and regression-based methods in at least five ways. First, configurational analysis mathematically draws upon Boolean algebra rather than linear algebra, an entirely different branch of mathematics. Second, configurational approaches report findings at the level of conditions rather than at the level of variables. Third, configurational analysis has a fundamentally different search target (Thiem, Baumgartner, & Bol, 2016). Whereas regression analytic methods quantify the strength of a relationship between variables, configurational analysis identifies necessary and sufficient conditions in what is known as a “minimal theory,” a unique combination of nonredundant conditions whose joint presence links directly to an outcome of interest (Baumgartner, 2008). Fourth, configurational approaches operate from a theoretical framework distinct from other quantitative approaches. Correlation- and regression-based methods, for example, draw upon an “interventionist” model, assessing the incremental effect of a unit difference in independent variable *X* on the values of dependent variable *Y*, controlling for all other variables. Configurational approaches, by contrast, rely on a “regularity” model, which states that *A* is a cause of *B* if and only if *A* is part of set of conditions *AX* that, *ceteris paribus*, is regularly followed by *B* (Baumgartner, 2008; Rohlfing & Zuber, 2019). Fifth, configurational approaches can analyze and model both causal complexity (i.e., the joint presence of conditions) and equifinality (i.e., multiple solution paths to the same outcome). This allows configurational methods to identify difference-making combinations of conditions, or in this case, elements of the OPTIMISTIC program or facility characteristics that distinguish one group of cases from another.

Configurational analysis, which includes specific methods like Qualitative Comparative Analysis (QCA) and Coincidence Analysis, is particularly well suited to quantitative cross-case analysis of nominal and ordinal data. In this analysis approach, numbers represent membership in a defined group rather than interval-level measures of a dimensional property, such as height, weight, or length. Results generated by configurational analyses go beyond identifying a list of “key” factors to explain both which conditions prove sufficient by themselves (e.g., sufficiency), and which conditions must combine with other conditions to get to the outcome (e.g., necessity). Configurational analysis provides a mathematical approach for searching across sets of factors to pinpoint difference-making conditions (Ragin, 1987; Thiem, 2017).

Configurational approaches have been applied in political science, sociology, and education for decades. In health research, it has become a part of the mixed-methods repertoire; configurational analysis, for example, was prominently featured in a 2019 Cochrane Review to identify conditions related to successful implementation (Harris et al., 2019), and highlighted in its own dedicated section in a 2019 review of innovations in mixed methods in public health (Palinkas, Mendon, & Hamilton, 2019). Configurational approaches remain relatively new to geriatric research. Published study protocols detail the use of configurational methods to help identify conditions linked to quality of life for older residents with dementia living in traditional and small-scale long-term care settings (A. H. P. M. De Rooij, Luijkx, Declerq, & Schols, 2010; A. H. De Rooij, Luijkx, Declerq, & Schols, 2011), as well as help develop and test a mobility and counseling program designed for older residents with dementia in a respite care setting (Heinrich, Cavazzini, & Holle, 2019). Configurational analysis has also been explicitly applied to examine how senior citizens’ self-efficacy, anxiety, self-reported health conditions, cognitive ability, and physical functioning influenced their perceptions of gerontechnology (Mostaghel & Oghazi, 2017) as well as to identify the relationship between patient-centered medical homes specializing in geriatrics and the quality of diabetes care (Thygeson et al., 2012).

The conceptual framework for this study was the Consolidated Framework for Implementation Research (CFIR; Damschroder et al., 2009). The CFIR framework provides an overall typology for understanding the implementation of interventions in health care. It describes five interrelated major domains (intervention characteristics, outer setting, inner setting, individual characteristics, and implementation process), and each domain has an additional 4–12 constructs attached to it. The CFIR Framework does not specify how these five domains and their associated constructs interrelate. Instead, the CFIR provides “a pragmatic organization of constructs upon which theories hypothesizing specific mechanisms of change and interactions can be developed and tested empirically” (Damschroder et al., 2009). The CFIR framework is both theory-based and evidence-based, and represents the accumulated result of more than 50 years of research on implementation and diffusion.

This configurational analysis used to evaluate the OPTIMISTIC model included factors from four of the five CFIR domains: characteristics of the intervention; the internal setting or organizational context in which the implementation occurs; the outer or external settings in which the organization exists; and the individuals involved in both the inner and outer setting who can promote implementation (Bauer et al., 2015). The implementation process was not included, as it was similar across nursing facilities. This methodology was employed to determine what conditions were present in nursing facilities that successfully reduced hospitalizations.

## Methods

### Setting

The OPTIMISTIC project (August 2012–September 2016) was implemented in 19 Indianapolis area nursing facilities between February and April 2013. It was deemed exempt from review by the Indiana University Institutional Review Board.

### Study Design

The OPTIMISTIC clinical model is implemented by specially trained Project RNs and NPs. Documentation of nurse activity regarding patient encounters is captured in the project database. Quarterly reports are submitted to CMS regarding participating resident enrollment and changes in facility leadership. Data from the Minimum Data Set 3.0 are used to verify enrollment reports. Additionally, project RNs completed quarterly facility engagement surveys to identify potential barriers to successful implementation and rate the engagement of senior leaders, including the director of nursing (DON) and executive director (ED).

### Participants

Nursing facility residents with a length of stay of 100 days or longer or no clear discharge plan were considered eligible for the initiative. Residents were excluded if they were enrolled in Medicare-managed care, per CMS requirements.

### Measures

The relevant and available data about conditions related to implementation effectiveness were conceptualized using the CFIR framework (Bauer et al., 2015; Table 1).

**Table 1.** Consolidated Framework for Implementation Research Domains and Study Data Elements

Domains	Data Elements
OPTIMISTIC intervention characteristics	• Rate of project RNs and NP clinical encounters per 1,000 resident days • Proportion of hospitalized residents who received a transition visit from the project NP on return to the facility • Proportion of residents who had engaged in advance care planning with a project RN
Inner setting—nursing facility characteristics	• Baseline rate of hospitalization prior to the start of the initiative • Project RN ratings of senior management (DON, ED) investment in the OPTIMISTIC mode • Severity of resident illness • CMS star ratings, a marker of overall facility quality
Individuals involved	• Turnover for the DON • Turnover for the ED • Project RN changes
Outer setting—external environment	• Medicare managed care penetration
Process of implementation	• Not included (consistent across sites)

*Note:* CMS = Centers for Medicare and Medicaid Services; DON = director of nursing; ED = executive director; NP = nurse practitioner; OPTIMISTIC = Optimizing Patient Transfers, Impacting Medical quality, Improving Symptoms—Transforming Institutional Care; RN = registered nurse.

#### Intervention Characteristics

The main intervention measures included in the analysis are the rate of project RNs and NP clinical encounters per 1,000 resident days, the proportion of hospitalized residents who received a transition visit from the project NP on return to the facility, and the proportion of residents who had engaged in advance care planning with a project RN. These variables are calculated using study enrollment data and the project database.

#### Nursing Facility Characteristics (Inner Setting)

Several variables were identified as potentially important characteristics of participating nursing facilities. These include the baseline rate of hospitalization prior to the start of the initiative, calculated using Minimum Data Set (MDS) data (January 2011–June 2012), severity of resident illness as assessed by the Changes in Health, End-Stage Disease and Symptoms and Signs (CHESS) scale (Ogarek, McCreedy, Thomas, Teno, & Gozalo, 2018), and CMS star ratings, a marker of overall facility quality. Senior management (DON, ED) investment in the OPTIMISTIC model was generated based on qualitative ratings provided by project RNs.

#### Individuals Involved

This variable was conceptualized as turnover for the DON, ED, and project RN. Changes in facility leadership were tracked as part of the quarterly reports to CMS. Changes in project clinical staff were tracked internally.

#### External Environment (Outer Setting)

Medicare-managed care penetration was tracked as part of the quarterly reports to CMS, as residents enrolled in these programs were excluded from OPTIMISTIC.

### Outcomes

The OPTIMISTIC clinical program was evaluated based on its success in reducing the hospitalization rates per 1,000 eligible resident days. There was facility variability in the level of reduction in hospitalizations. For analysis purposes, the target outcomes were relative declines of greater than 10% and 20% in all-cause hospitalizations per 1,000 eligible resident days. The 10% and 20% reduction thresholds were selected because they represented meaningful cutoffs in terms of quality improvement for OPTIMISTIC. Eligible residents and hospitalizations overlapping the +101 day period of the stay were identified from MDS data and divided into two 18-month observation periods: pre-OPTIMISTIC (January 1, 2011–June 30, 2012); and full OPTIMISTIC intervention (January 1, 2015–June 30, 2016). Percent decline was calculated comparing pre-OPTIMISTIC and full OPTIMISTIC periods.

### Data Analysis

#### Data Reduction

Our first objective was to reduce the data set. Theoretical and empirical knowledge identified the original 11 factors—consisting of six dichotomous factors and five multivalue factors—as potential explanatory factors. We used the “msc” function in the Coincidence Analysis package (Ambühl & Baumgartner, 2019; “cna”) in R to consider fully all 11 potential explanatory factors and 19 cases at once in order to identify the configurations of conditions with the strongest connection to the target outcomes (i.e., greater than 10% and 20% decline in hospitalizations per 1,000 resident days). The consistency cutoff for inclusion was initially set at 100% and then reduced by decrements of 5% if no configurations met the consistency threshold. We set “r” to a maximum of 4, where r stands for the number of objects to be selected at the same time from a larger set of *n* objects (i.e., the 11 potential explanatory factors). In setting r to a maximum of 4, we considered all one-, two-, three-, and four-condition configurations across the 11 factors that were instantiated within the data set and met the cutoff threshold. We then ranked these configurations by coverage, looking for configurations that explained the greatest number of cases at the selected consistency level (e.g., 100%): high coverage scores suggested strong connections between specific conditions and the target outcome (Yakovchenko et al., 2020). We then assessed this condition-level output on the basis of our research question (i.e., at least one program-related condition had to be present) as well as logic, theory, and prior knowledge to narrow the initial set of 11 potential explanatory factors to a smaller subset of candidate factors to model. Within this subset, we converted any multivalue factors to dichotomous factors in order to reduce dimensionality, using cutoffs based on theoretical grounds as well as on critical thresholds identified in the “msc” condition-level output. For example, the multivalue factor for leadership commitment was originally multivalue, but it made both theoretical and empirical sense to code “strongly agree” or “agree” as 1, and “neutral” or “disagree” as 0, as only “strongly agree” and “agree” had a strong connection to the outcome. Likewise, with the multivalue factor for baseline hospitalization rates, it made both theoretical and empirical sense to distinguish the top performers (i.e., those in the top quartile with the lowest initial hospitalization rates) from everyone else, as facilities not in this top performance quartile at baseline were the facilities with the higher hospitalization rates at baseline and thus had the biggest room for improvement. Table 2 contains the analytic data set used in the configurational analysis.

**Table 2.** Analytic Data Set With Outcomes and Conditions for Facility-Level OPTIMISTIC Factors Included in the Configurational Analysis

Facility ID	Outcomes				Conditions			
	Hospitalization Rate Before Program^a^	Hospitalization Rate During Program^b^	10% Decline or Greater^c^	20% Decline or Greater^d^	Director of Nursing Turnover^e^	Senior Management Support^f^	Baseline Hospitalization^g^	CHESS^h^
1	3.146	1.251	1	1	0	1	0	1
2	3.270	1.410	1	1	1	1	0	1
3	4.372	1.711	1	1	1	1	0	1
4	1.922	1.262	1	1	0	1	0	1
5	2.670	1.960	1	1	0	1	0	0
6	2.417	1.219	1	1	1	1	0	0
7	1.530	1.196	1	1	0	1	0	1
8	1.690	1.224	1	1	1	1	0	0
9	1.079	0.803	1	1	1	1	1	1
10	2.908	1.830	1	1	0	0	0	0
11	2.214	1.443	1	1	1	0	0	1
12	1.537	1.155	1	1	1	0	0	1
13	1.734	1.202	1	1	0	0	0	1
14	1.331	1.147	1	0	1	0	1	0
15	3.422	3.053	1	0	1	0	0	0
16	0.963	1.137	0	0	0	1	1	1
17	0.749	0.831	0	0	0	1	1	0
18	0.906	1.528	0	0	0	1	1	1
19	1.823	2.335	0	0	0	0	0	1

*Note:* CHESS = Changes in Health, End-Stage Disease and Symptoms and Signs; OPTIMISTIC = Optimizing Patient Transfers, Impacting Medical quality, Improving Symptoms—Transforming Institutional Care.

^a^Number of hospitalizations per 1,000 resident days for the period January 2011 through June 2012.

^b^Number of hospitalizations per 1,000 resident days for the period January 2015 through June 2016.

^c^10% decline or greater in hospitalization rate comparing pre and during program periods (1 = achieved ≥10% decline, 0 = did not achieve ≥10% decline).

^d^20% decline or greater in hospitalization rate comparing pre and during program periods (1 = achieved ≥20% decline, 0 = did not achieve ≥20% decline).

^e^Director of nursing turnover during observation period (1 = 1 or more turnovers, 0 = no turnover).

^f^Project nurse (RN) response to “senior management supports program through investment in resources”; average within facility rating (1 = *agree or strongly agree*, 0 = *neutral or disagree*).

^g^Number of hospitalizations per 1,000 resident days for the period January 2011 through December 2012 (1 = above 25th percentile, 0 = below 25th percentile).

^h^Average CHESS score (1 = *1 or more*, 0 = *less than 1*; possible CHESS scores range from 0 = *most stable* to 5 = *least stable*).

#### Model Development

We proceeded to model development with this subset of dichotomous factors, where we applied “crisp-set” QCA using the R package “QCApro” (Thiem, 2018). We began by setting the consistency threshold to 100%, then lowered it by decrements of 5% as needed. As part of our evaluation criteria, we looked for models where the same set of factors could explain both target outcomes (i.e., greater than 10% and 20% decline in hospitalizations per 1,000 resident days) with ≥80% consistency and ≥80% coverage, where each term in the model had ≥15% unique coverage, with no model ambiguity. Final models were developed using QCApro.

## Results

Initial data reduction using the “msc” routine was achieved at 100% consistency; it was not necessary to lower the consistency level further in order to identify a subset of candidate factors to model.

### Outcome 1: Greater Than 10% Decline in Hospitalizations

**Figure 1.**
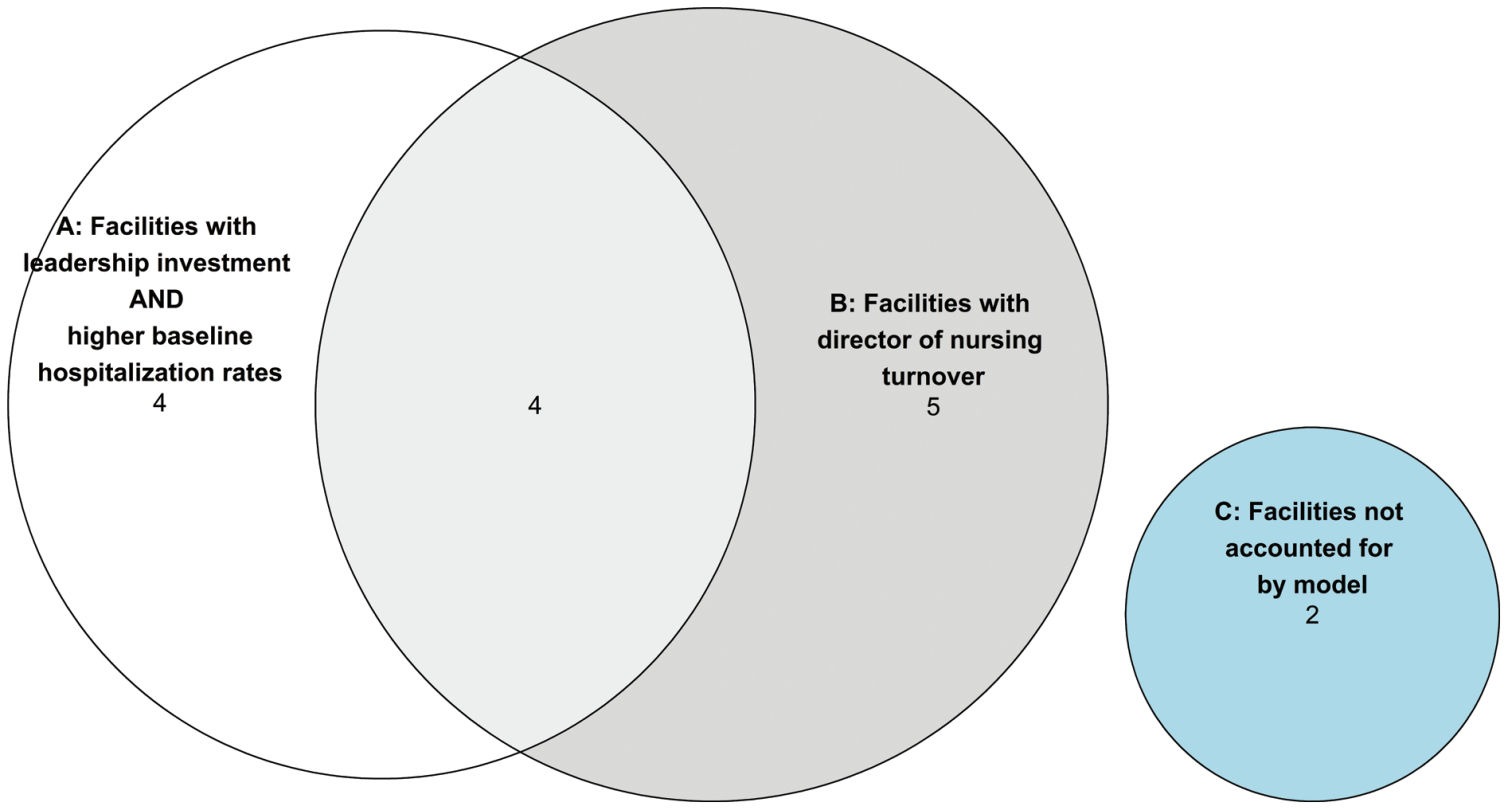
Solution visualization for the Optimizing Patient Transfers, Impacting Medical quality, Improving Symptoms—Transforming Institutional Care facilities (*n* = 15) that had a ≥10% decline in hospitalization rates.

Using the subset of factors identified in data reduction, configurational analysis identified a three-condition solution that identified facilities with >10% decline in hospitalizations with 100% consistency (13/13). The solution had one program-related condition and two organizational-related conditions with 87% coverage (13/15; Figure 1). The three conditions were: (a) leadership investment as rated by project RN; (b) higher baseline rates of hospitalization prior to the start of the initiative (e.g., greater room for improvement at baseline); and (c) Director of Nursing turnover during the observation period. In plain language, this model had two solution paths:

- the combination of leadership investment in the program AND higher baseline hospitalization rates; OR
- turnover in the director of nursing (DON).

The solution is visually represented in Figure 1. There were 15 facilities of the original 19 facilities that exhibited the outcome of 10% decline in hospitalization rates. Among the 15 facilities that exhibited a decline of >10% in the hospitalization rate, there were four facilities covered by the first solution path only, five facilities covered by the second solution path only, and four facilities covered by both solution paths. Two facilities were unaccounted for by the model (C) yielding a coverage rate of 0.87 (i.e., 13/15). There were no “necessary” conditions for the ≥10% improvement outcome, given that the two paths to the outcome featured different conditions. A change in the DON position, though, proved “sufficient”; all nine facilities with this condition present achieved the ≥10% improvement outcome.

### Outcome 2: Greater than 20% Decline in Hospitalizations

**Figure 2.**
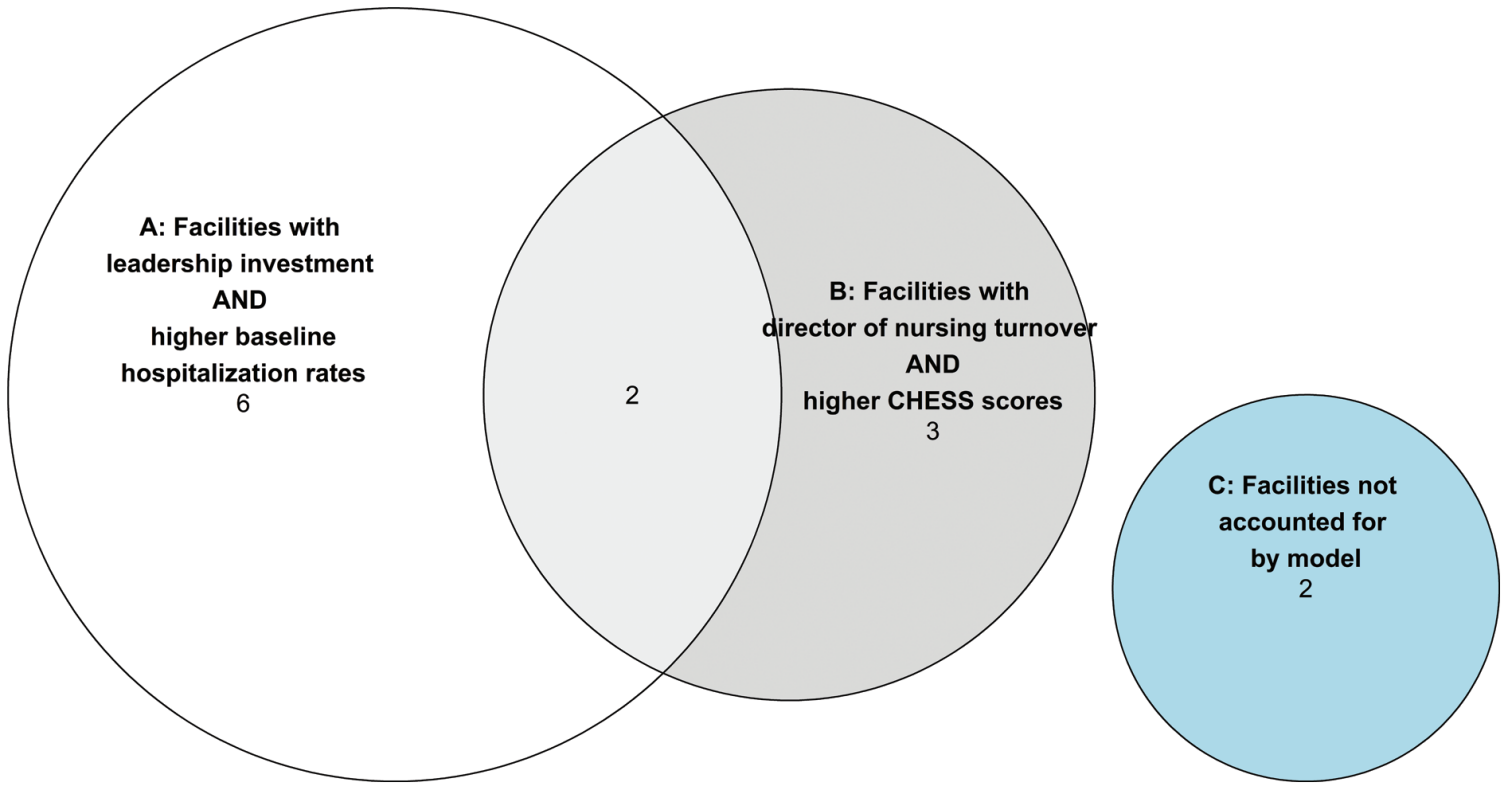
Solution visualization for the Optimizing Patient Transfers, Impacting Medical quality, Improving Symptoms—Transforming Institutional Care facilities (*n* = 13) that had a ≥20% decline in hospitalization rates.

For the outcome of 20% decline in hospitalizations, configurational analysis identified a four-condition solution that identified facilities with 20% or greater decline in hospitalizations, again with 100% consistency (11/11). The solution had one program-related condition and three organizational-related conditions and had 85% coverage (11/13; Figure 2). The four factors included the same three factors in the 10% decline model plus a condition indicating CHESS score (a measure of acuity). The model had two solution paths:

- the combination of leadership investment in the program AND higher baseline hospitalization rates; OR
- turnover in the director of nursing AND a CHESS score of 1 or higher (i.e., higher acuity).

There were 13 facilities of the original 19 facilities that exhibited the outcome of 20% decline in hospitalization rates. Among these 13 facilities that exhibited a decline of 20% or more in the hospitalization rate, there were six facilities covered by the first solution path only, three facilities covered by the second solution path only, and two facilities covered by both solution paths. This model had no single “necessary” or “sufficient” conditions, as each of the two solution paths required that two different conditions appear together. Two facilities were unaccounted for by the model (C) yielding a coverage rate of 0.85 (i.e., 11/13).

## Discussion

The overall OPTIMISTIC clinical demonstration project had an overall positive impact on reducing hospitalizations of long-stay nursing facility residents. The results of this analysis provide additional information about the barriers, facilitators, and conditions associated with more successful outcomes from the OPTIMISTIC model. The combination most directly connected with a 10% reduction in hospitalization rates involved one program-related factor and two organizational factors: (a) the investment of resources by senior management; (b) ample room for improvement in terms of levels of hospitalizations at baseline; or (c) any turnover in the facility director of nursing. The combination of factors most directly connected with a 20% decline was similar, but also suggested the model was more successfully implemented in facilities with residents who were less medically stable as measured by a CHESS score. Some of these findings are intuitive and consistent with both the literature and general tenets of successful implementation of new programs, such as investment of senior management, whereas other are more unexpected.

It is unsurprising that facilities with higher baseline rates of hospitalization experienced better outcomes once attention and resources were focused on reducing hospitalizations. It is likely that facilities with higher rates of baseline hospitalizations had a greater proportion of potentially avoidable hospitalizations that could be more safely managed in house. The most common causes of potentially avoidable hospitalizations include pneumonia, urinary tract infection, skin ulcers, dehydration, chronic obstructive pulmonary disease/asthma, and congestive heart failure (Spector, Limcangco, Williams, Rhodes, & Hurd, 2013). The multicomponent OPTIMISTIC clinical model (Unroe et al., 2015) targeted these common conditions and enhanced care overall through proactive assessment and direct clinical care.

Our finding that investment by senior management is linked with successful outcomes is consistent with prior research identifying associations between leadership and the adoption of change (Castle & Decker, 2011; Corazzini et al., 2015; Donoghue & Castle, 2009). Although the measurement in this study focused specifically on leadership’s willingness to invest resources in the adoption and implementation of a new clinical model, it is likely that this is associated with high-quality leadership characteristics that facilitate culture change, such as open communication, shared decision-making, staff education, and a focus on positive leadership–staff relationships (Miller et al., 2018). In a prior qualitative study of barriers to implementation of OPTIMISTIC, we found that miscommunication was a major barrier to implementation which often resulted in a lack of knowledge about both the OPTIMISTIC program and how to interact with program staff (Ersek, Hickman, Thomas, Bernard, & Unroe, 2018). This finding reinforces our understanding of what is necessary in order to create change within the nursing facility setting.

Although it may seem surprising that DON turnover was also a factor associated with successfully reducing hospitalization rates, others have found similar outcomes. In a 2010 study, Castle and Lin also found that DON turnover was associated with improved quality, hypothesizing that new nurses may be motived to improve clinical quality and view this as a top priority (Castle & Lin, 2010). DON turnover could represent the departure of an unsupportive DON who was resistant to the changes required to implement new programs such as OPTIMISTIC (Ersek et al., 2018). It may also be an opportunity for the OPTIMISTIC RN to exert more influence in implementing the OPTIMISTIC model, as a new DON hired with the expectation of partnership may be more open to collaborate with the Project RN. New DONs may be more likely to view the partnership with the demonstration project and goal of reducing potentially avoidable hospitalizations as a normal part of the role, rather than an add-on that requires additional effort or changes in existing practice. Finally, it is also possible that facilities provide additional resources at the time of DON turnover to assist during the transition period.

Facilities with sicker, less-stable populations as reflected by higher CHESS Scores also experienced greater hospitalization reduction. Similar to the finding that facilities with higher baseline hospitalization rates had greater reductions, it may be that facilities with sicker residents have greater opportunity to reduce hospitalizations. Additionally, the addition of skilled clinical staff, with a proactive approach to chronic disease management, may have been particularly impactful in these facilities. Finally, residents and families in OPTIMISTIC facilities have access to high-quality advance care planning—many residents with advanced disease when given the opportunity for a robust goal of care decision will choose to limit interventions including hospital transfer (Hickman et al., 2016, 2019).

### Limitations

There are several limitations to this work. First, the analysis relied on observational data collected as part of OPTIMISTIC clinical demonstration project, not specifically for this analysis. Secondly, although we were able to include variables for four of the five CFIR domains, there are multiple factors that could have impacted implementation that were not included. For example, no information was collected about intensity of medical coverage by primary care medical providers, which may be associated with a population with higher acuity and influence success in an intervention to reduce hospital transfers. Moreover, it was outside the scope of this demonstration project to capture information about staffing or operational issues such as communication. Finally, configuration–outcome connections are not inherently causal. While there is ample evidence for our solution pathways, which meet consistency and coverage requirements and are consistent with logic, theory, and prior knowledge, replication and experimental work is ultimately needed to establish the strength of the causal relationship between specific conditions and improvement outcomes.

## Conclusions

The OPTIMISTIC clinical demonstration project had an overall positive impact on reduction of hospital transfers, compared with control group nursing facilities in the state (Ingber et al., 2017). Although the external evaluation determined that the overall model was successful, this finding reflects a range of experiences within the 19 different nursing facilities participating in OPTIMISTIC. These analyses provide further descriptions of factors associated with the highest performers in the demonstration. Directions for future research include examining the effect of staffing ratios and the quality of clinical care on outcomes. Findings suggest organizations interested in adopting models to reduce potentially avoidable hospitalizations like OPTIMISTIC target facilities with higher baseline hospitalization rates where there is greater room for improvement. Senior leadership needs to be strongly committed to implementation and act in ways that support adoption of new practices. Finally, it may be important to consider a switch in clinical leadership as an opportunity (rather than as a detriment) for enhancing implementation and culture change by virtue of bringing in new perspectives and viewpoints.

## Funding

OPTIMISTIC is supported by the Centers for Medicare and Medicaid Services (CMS) of the U.S. Department of Health and Human Services (HHS) as part of an award totaling $16,545,692 with 0% percentage financed with nongovernmental sources. The contents are those of the author(s) and do not necessarily represent the official views of, nor an endorsement, by CMS, HHS, or the U.S. Government.

## Conflict of Interest

None reported.
